# ChatGPT-Delivered Physical Activity Intervention for Children With Autism Spectrum Disorder: Pre-Post Feasibility Study

**DOI:** 10.2196/71119

**Published:** 2025-06-11

**Authors:** Uğur Aydemir

**Affiliations:** 1 Faculty of Sport Sciences, Bayburt University, 26 Muteber Street, Bayburt, 69000, Turkey, 90 5534935490

**Keywords:** autism spectrum disorder, physical activity, parent, ChatGPT, child

## Abstract

**Background:**

The use of digital technologies, such as mobile apps, Zoom (Zoom Communications), virtual reality, and video games, to promote physical activity in individuals with autism spectrum disorder (ASD) has been increasing. However, there are no studies using ChatGPT (OpenAI), a popular tool in recent years, for promoting physical activity in children with ASD.

**Objective:**

This study aimed to evaluate the feasibility and potential effectiveness of ChatGPT-delivered physical activity interventions in children with ASD.

**Methods:**

A total of 26 families (parent-child dyads) participated in the study. Families were randomly assigned to an application group (n=13) and a control group (n=13). In the application group, parents implemented physical activities recommended by ChatGPT for their children with ASD. Data were collected using the Leisure Time Exercise Questionnaire (LTEQ) and a feasibility questionnaire.

**Results:**

Parents reported that ChatGPT-delivered physical activities were a feasible intervention to increase physical activity levels in children with ASD. They also found the activity content suggested by ChatGPT to be interesting and useful. LTEQ measurements corroborated these findings, showing a significant increase in the physical activity levels of children in the intervention group after the intervention.

**Conclusions:**

The results suggest that ChatGPT-delivered physical activities could be a promising intervention to enhance physical activity in children with ASD. Further investigation is warranted.

## Introduction

Autism spectrum disorder (ASD) is a neurodevelopmental disorder that commonly emerges in early childhood and is characterized by the deficiencies in social interaction and communication skills and the restricted-repetitive behavioral patterns [1]. ASD negatively affects the behavioral, social, cognitive, and mental functions of children diagnosed with this disorder [2], as well as their physical activity levels [3]. Recent studies have revealed that children with ASD are less physically active than their typically developing peers [4], and that these children tend to lead sedentary lives [5]. Many individuals with disabilities, including children with ASD, spend a lot of time in sedentary behaviors such as watching television [6]. High levels of inactivity, a concern for all children, is an important issue to address since children with ASD have fewer opportunities to participate in physical activity [7,8].

Participation in physical activity provides many benefits to children with ASD in terms of physical and mental health [9-11]. Studies examining physical activities in children with ASD have revealed that physical activities can have a positive effect on manipulative and locomotor skills [12-14]. At the same time, some studies have found that physical activity helps increase the self-confidence and self-efficacy of children with ASD [10,14]. Furthermore, some studies have shown that stress and anxiety levels of children with ASD, who participate in physical activity, decrease [15] while their social and communication skills improve [16,17].

Despite the numerous health and social benefits of regular physical activity, there are many potential barriers that limit the participation of children with ASD in physical activity [9]. To explain the barriers that cause physical activity deficiencies in children with ASD, some studies focus on characteristic features of ASD, such as inadequate motor skills (joint flexibility, balance, speed, etc), biophysical behaviors (inattention and hyperactivity), and communication disorders [18-20]. Other studies have drawn attention to the limitations in the sustainability of current physical activity interventions for children with ASD [21]. Current physical activity interventions aimed at increasing the physical activity level of children with ASD often require specialist support (teacher, medical clinician, or physiotherapist), costly specialized equipment, and unnatural environments (school, horse, pool, or equine therapy center, etc) [21]. In most cases, access to existing physical activity programs is limited to people living in areas where specialists provide services [22], and especially during global crises such as COVID-19 pandemic, children with ASD may have limited access to physical activity [3]. For this reason, it is stated that there is a need for an alternative physical activity presentation that children with ASD can do at home with their parents and reach a wider audience.

Recent studies have suggested that internet technologies, programs, and apps may have significant potential in promoting physical activity in children with ASD and creating a more sustainable physical activity presentation [21,23,24]. Among these apps, conversational agents using large language models (LLMs), which have recently become widely used worldwide, have attracted attention as powerful information search tools for children with ASD [25]. Their ability to produce contextually relevant and consistent text positions conversational agents using LLM as potential agents, especially for educational content [26]. Extensive information can be easily accessed anytime and anywhere by simply typing a query about individuals with ASD into conversational agents, usually exemplified by ChatGPT (OpenAI) [27,28]. Conversational agents using LLM provide useful information about daily living for individuals with ASD, such as daily tasks, personal care, and social interaction [29,30].

Although there are different conversational agents such as ChatGPT, BERT (Google), Cipherbot, and DeepSeek in the literature, it has been seen that ChatGPT is frequently used for children with ASD. These studies [31-35] included important information about application protocols and ChatGPT prompts. For this reason, this study used ChatGPT to deliver physical activities to parents of children with ASD. Released by OpenAI in November 2022, ChatGPT is an open AI language model that produces human-like responses to text-based prompts [36]. ChatGPT, which can run on a phone, tablet, or computer, can understand responses in a variety of languages, write stories of different types and lengths, summarize information in complex texts, provide explanations on various topics, and even refuse to respond to inappropriate prompts [37]. ChatGPT is also a highly advanced app in that it can provide a continuous dialogue by remembering what the user has said before in the conversation thread [38]. Although limited in number, studies [31-35] have revealed that ChatGPT can be a useful tool for improving the social skills of children with ASD or for providing information to parents about children with ASD. Although it is a new topic, there is no study evaluating the effectiveness and feasibility of ChatGPT-delivered physical activities in children with ASD. Considering this gap in the literature, the aim of this feasibility study was to examine the effectiveness and feasibility of ChatGPT-delivered physical activities to increase the physical activity level of children with ASD.

## Methods

### Study Design

This study was designed to evaluate the feasibility of ChatGPT-delivered physical activities to increase the physical activity level of children with ASD. A feasibility study examines whether an intervention is suitable for further testing [39] and allows the intervention procedure to be used in future studies [21]. The feasibility process of the study was based on previous studies that used feasibility measures to evaluate a web-based intervention [21,22,24,40-42] and consisted of 5 stages (Textbox 1).

Textbox 1. **Feasibility process of ChatGPT-delivered physical activity.**

- Stage 1 (assessment for participant eligibility): The researcher collaborated with a local special education and rehabilitation center in Ankara to recruit parents of children with autism spectrum disorder as participants.
- Stage 2 (ChatGPT training for parents): Parents attended three 40-minute training sessions that included information about ChatGPT and physical activity.
- Stage 3 (preintervention measurements): A week before the intervention, the physical activity level of children with autism spectrum disorder.
- Stage 4 (intervention [4 weeks]): Parents practiced the physical activities recommended by ChatGPT for 40 minutes, 3 days a week for 4 weeks.
- Stage 5 (postintervention measurements): A week after the intervention, the physical activity level of children with autism spectrum disorder and parents’ opinions on ChatGPT-delivered physical activities were evaluated.

### Participants

The participants of the study were recruited from Ankara province. The criterion sampling method was used in determining the participants [43]. Inclusion criteria were individuals agreeing to participate in the study voluntarily, having a child diagnosed with ASD according to the *Diagnostic and Statistical Manual of Mental Disorders, Fifth Edition* criteria, and parents and children not having any health problems that prohibit physical activity. In line with these criteria, support was received from a special education and rehabilitation center to identify parents. With the permission of the director of the association, the primary researcher interviewed the participant candidates on the phone and explained the aim of the study and participation process to them. Consent forms were received from all parents who voluntarily participated in the study via email. A total of 26 parents (9 mothers and 17 fathers) and their children with ASD participated in the study. All parents filled out the demographic information form and physical activity questionnaire (pretest) before the intervention and the feasibility measures and physical activity questionnaire (posttest) after the intervention. The characteristics of the parents and their children with ASD are presented in Table 1.

**Table 1.** The characteristics of the participants.

Characteristics	Groups	
	Application (n=13)	Control (n=13)
Children (n=26)		
Sex, n (%)		
Female	6 (46)	8 (62)
Male	7 (54)	5 (38)
Age (years), mean (SD)	14.38 (3.06)	13.92 (3.52)
Diagnosis, n (%)		
ASD^a^	13 (100)	13 (100)
Additional comorbidities, n (%)		
SD	1 (8)	1 (8)
ID	11 (84)	7 (54)
N	1 (8)	5 (38)
Parents (n=26)		
Gender, n (%)		
Female	5 (38)	4 (31)
Male	8 (62)	9 (69)
Age (years), mean (SD)	42.84 (7.70)	44.07 (7.95)
Education, n (%)		
Secondary school	5 (38)	3 (23)
High school	4 (31)	7 (54)
University	4 (31)	3 (23)
Income, n (%)		
Low	3 (23)	—^b^
Middle	7 (54)	8 (62)
High	3 (23)	5 (38)

a ASD: autism spectrum disorder.

b Not available.

### Intervention

#### ChatGPT Training for Parents

In this study, parents played an important role by actively participating in ChatGPT-delivered physical activities and helping their children with ASD. Parental participation was an integral part of ChatGPT-delivered physical activities. To prepare parents for this role, the researcher conducted three 40-minute training sessions. Each session lasted until parents demonstrated proficiency. The first session focused on developing parents’ skills in using ChatGPT. The researcher explained to parents how to download ChatGPT and how to get physical activity recommendations using ChatGPT. Then, the researcher asked all parents to give ChatGPT the following command: “Create a 4-week, 3-day, 40-minute physical activity program that can be implemented at home for my child who is…years old and has ASD.” The researcher discussed the program created by ChatGPT with parents and told them to give the following command to ChatGPT for physical activities they did not understand: “What is…activity? Show me in detail.” The researcher emphasized the importance of all parents using the commands suggested by the researcher in order to optimize the prompts. The second session included informing parents about the parts of a physical activity (warm-up, cool-down, and main part). The third session addressed strategies that parents can use when providing physical activity to children with ASD. These strategies generally included information about preparing children with ASD for physical activities (wearing sports clothes, social stories, and preview of activities and equipment), organizing the environment where physical activities will take place (safety, ventilation of the environment and sports pictures), including other members of the family in physical activities (parent and sibling participation), and reward is given. A special WhatsApp (Meta) group was created to answer parents’ questions during the implementation of the ChatGPT-delivered physical activities. Parents’ questions regarding the implementation of the physical activities and the use of the ChatGPT software were answered instantly via the WhatsApp group. In addition, at the end of the daily physical activity session, parents chatted in the WhatsApp group to evaluate the effectiveness of the session and explore the experiences of other parents.

#### ChatGPT-Delivered Physical Activities

All parents, who completed the ChatGPT training, used ChatGPT-delivered physical activities to increase the physical activity levels of their children with ASD. One week before the intervention, the researcher asked each parent to give ChatGPT an appropriate command so that the physical activities were age-appropriate for children with ASD and could be easily carried out in the home environment. Although the content of ChatGPT varied, it generally suggested physical activities to parents consisting of 3 parts: warm-up, main part, and cool-down (Table 2). The researcher asked parents to ask ChatGPT again about the activities they did not understand about the activities suggested by ChatGPT until the intervention began. In addition, the researcher supported the parents by explaining the activities they did not understand in the WhatsApp group. The parents implemented the intervention for 40 minutes, 3 days a week for 4 weeks. The parents and the children with ASD participated in the activities together. During the implementation process, the researcher immediately answered the parents’ questions about the activities and strategies in the WhatsApp group. In addition, at the end of the daily physical activity session, the parents chatted in the WhatsApp group to evaluate the effectiveness of the session and prepare them for the next session. In the interview, the researcher asked the parents questions about the extent to which physical activities were performed, who participated in physical activities, and what the benefits of physical activities were. In this way, the researcher tried to verify to what extent and how physical activities were carried out. All parents and children with ASD completed the 4-week intervention. The recommended physical activities in a typical week are shown in Table 2.

**Table 2.** Physical activities in a typical week recommended by ChatGPT.

Day and physical activity	Content
First day (movement and balance)	
Warming up (10 min)	Opening and closing the arms up and to the sides; slow walking with the knees pulled in; slow running in place.
Main exercise	
Balance line (5 min)	Draw a line on the ground and have your child walk along this line.
One leg on pillow (5 min)	Try to keep your balance on the pillow.
Rolling with ball (5 min)	Sit on the floor with your child and roll a large ball to each other.
Jumping (5 min)	Use a ring or cushion to bounce in and out.
Cooling down (10 min)	Slow walking and stretching movements (stretching the arms and stretching the legs).
Second day (coordination and movement)	
Warming up (10 min)	Stretching arms and legs; animal walks (bear walk, frog leap).
Main exercise	
Throwing and catching balls (10 min)	You and your child throw and catch a large ball to each other.
Rolling on the mat (5 min)	Rolling back and forth on a large cushion.
Imitation games (5 min)	Exercises of standing on one leg and finding balance while turning slightly.
Cooling down (10 min)	Slow breathing and yawning.
Third day (game day and free movement)	
Warming up (10 min)	Free dance and simple movements with rhythmic music.
Main exercise	
Balloon games (10 min)	Play a game with your child by holding balloons in the air.
Obstacle course (10 min)	Set up a simple track with cushions and chairs inside the house and have your child walk through the track (crawling, walking, and jumping).
Cooling down (10 min)	Slow paced walking and light stretching.

### Data Collection

#### Overview

Data were obtained with the following 2 tools: (1) Feasibility Questionnaire and (2) Leisure Time Exercise Questionnaire (LTEQ). Measurements were made 1 week before and after the intervention. Data were collected via Google forms. The researcher shared the Google form links in the WhatsApp group and asked all parents to complete the forms in the links within 1 week. A personal information form was used to obtain information about the demographic characteristics of parents (age, gender, and level of education) and children with ASD (age, gender, additional comorbidities, and number of people in the household).

#### Feasibility Questionnaire

Parents rated their ChatGPT-delivered physical activities via the Feasibility Questionnaire after the 4-week intervention. Questionnaire questions were designed considering previous studies investigating the physical activity level of children with ASD [3,21]. Questionnaire questions included the following: (1) how would you rate your overall experience with ChatGPT-delivered physical activities, (2) how interesting were the ChatGPT-delivered physical activities, (3) how beneficial were the ChatGPT-delivered physical activities for your child’s physical activity level, and (4) to what extent did you learn about physical activity through ChatGPT? The Feasibility Questionnaire was rated on a 5-point Likert-type scale (very satisfied to not very satisfied, very to a little, very useful to not useful at all).

#### Leisure Time Exercise Questionnaire

To assess the physical activity level of children with ASD before and after the intervention, the researcher used the LTEQ developed by Godin and Shephard [44]. The LTEQ has been frequently used in previous studies [3,21,22,45-47] to determine the leisure time physical activity level of individuals with ASD. Memari et al [46] showed that the LTEQ has a good test-retest reliability score that can be used to determine the physical activity level of children with ASD. The questionnaire includes questions about at least 15 minutes of leisure time physical activity performed in the last 7 days and aims to determine the number of times of strenuous-intensity physical activities, moderate-intensity physical activities, and mild-intensity physical activities performed in the last week. To calculate the total score of the scale, high-intensity activities are multiplied by 9, moderate-intensity activities by 5, and light activities by 3, and all are added together. The formula is as follows: weekly leisure time activity score = (9 × strenuous intensity) + (5 × moderate intensity) + (3 × mild intensity). The calculated values are added up and generally evaluate the individual’s leisure time activity. In this evaluation, scores of 24 and above are classified as “Active,” 14‐23 as “Moderately active,” and 13 and below as “Not sufficiently active” [48]. The Turkish adaptation study of the LTEQ was conducted by Yerlisu-Lapa et al [49]. As a result of the exploratory factor analysis conducted to determine the factor structure of the questionnaire, the total correlations of the items were determined as 0.80, 0.76, and 0.65 for all 3 items, respectively, and it was found that 55% of the total variance was explained and gathered under a single factor. Test-retest reliability analysis yielded a Pearson correlation coefficient of r = 0.84 for the entire Leisure-Time Exercise Questionnaire (LTEQ), and 0.80, 0.76, and 0.72 for its vigorous, moderate, and mild intensity subscales, respectively. To assess equivalent form reliability, a comparison was made between the LTEQ and the International Physical Activity Questionnaire–Short Form (IPAQ-SF). The Spearman correlation coefficient for the relationship between the two forms was found to be ρ = 0.92, indicating a strong positive correlation [49].

### Statistical Analysis

Descriptive statistics (mean, SD, percentage, and frequency) were used in the study for feasibility measurements, demographic information, and frequency of participation in physical activities. A 2-way mixed ANOVA (analysis of variance; 2 groups × 2 time points) was used to determine the effects of ChatGPT-delivered physical activities (from baseline to intervention) on the physical activity level of children with ASD. All these statistical processes were carried out with the SPSS package program (version 25.0; IBM Corp).

### Ethical Considerations

The research was approved by the Bayburt University noninterventional clinical research ethics committee (number 209) on July 23, 2024. This study was approved by the Ethics Committee of Bayburt University (no. E-15604681-100-215816). Informed consent was obtained from the legal guardians of the participants. Participation was voluntary, and the participants had the right to withdraw from the study at any time. For secondary analyses using existing data with primary consent, the original consent or institutional review board approval covers secondary analyses without additional consent. Data have been anonymized and deidentified (pseudonyms were used in the study to protect the identities of the participants. Participant names were listed as P1, P2, P3, and so on. No financial compensation was given to the participants, but small symbolic rewards (stickers, reward cards, etc.) were given.

## Results

### Parental Opinions and Feedback on Feasibility of ChatGPT-Delivered Physical Activity

All parents expressed that they were very happy to participate in the ChatGPT-delivered physical activities. According to the parents, the ChatGPT-delivered physical activity content was interesting and very useful. Parents stated that the ChatGPT-delivered physical activities positively affected the physical activity levels of their children with ASD. Parents reported that they gained important information about physical activity through ChatGPT. In total, 92% (12/13) of the parents participated in the discussions in the WhatsApp group and shared pictures and videos of their children in the group.

### Physical Activity Level

In order to determine the effect of ChatGPT-delivered physical activities on the physical activity level of children with ASD, a 2-way mixed ANOVA (2 groups × 2 time points) test was performed (Table 3). While the mean physical activity score of children with ASD in the application group was 6.69 (not sufficiently active) before the intervention, their physical activity score increased to 34 (active) after the intervention. In contrast, while the mean physical activity score of children with ASD in the control group was 7.46 (not sufficiently active) before the intervention, their physical activity score decreased to 7.23 (not sufficiently active) after the intervention. Regarding the mean physical activity scores, 2-way mixed ANOVA revealed a significant group effect (*F*_1, 24_=108.769; *P*<.05, *ηp^2^*=.819), a significant time effect (*F*_1, 24_=341.333; *P*<.05, *ηp^2^*=.934) and a significant group × time interaction (*F*_1, 24_=353.069; *P*<.05, *ηp^2^*=.936) on the physical activity level of the participants in the application and control groups.

**Table 3.** Analysis of variance results related to the pretest-posttest physical activity scores.

Source	Type III sum of squares	Mean square	*F* test (*df*)	*P* value	Partial eta squared (ηp^2^)
Between-Subjects	9969.231				
Group (A/C)	2197.000	2197.000	108.769 (1, 24)	<.001^a^	.819
Error	484.769	20.199	—^b^	—	—
Within-subjects	5014.999				
Time (pretest-posttest)	2382.769	2382.769	341.333 (1, 24)	<.001^a^	.934
Time group	2464.692	2464.692	353.069 (1, 24)	<.001^a^	.936
Error	167.538	6.981	—	—	—

a Significant at *P*<.05.

b Not applicable.

**Figure 1.**
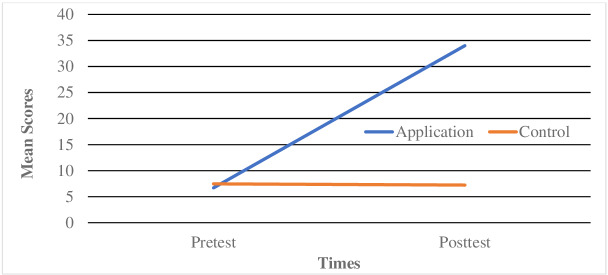
Mean physical activity scores of the groups in preintervention and postintervention.

Since ANOVA results reveal a significant group × time interaction, the adjusted Bonferroni value was used to determine whether there was a change in application and control groups over time. As reported in Figure 1, pair-wise comparisons revealed the significant increase in the preintervention physical activity scores of children with ASD in the application group compared with their postintervention physical activity scores (adjusted Bonferroni*: P*<.05, difference: +27.308); this was not the case in the control group (adjusted Bonferroni*: P*>.05, difference: −0.231).

## Discussion

### Principal Findings

The aim of this study was to examine the effects of ChatGPT-delivered physical activity intervention on the physical activity level of children with ASD. The results revealed that there was a positive and significant increase in the physical activity level of children with ASD in the application group after ChatGPT-delivered physical activities compared with children with ASD in the control group. Current physical activity interventions that aim to increase the physical activity level of children with ASD generally include physical activity programs such as karate [50], swimming [51], summer camp [52], fitness [53], and therapeutic horseback riding [54]. Systematic reviews suggest that participation in existing physical activity programs has a positive effect on the developmental domains of children with ASD [10,12]. Despite these promising findings, there are some limitations in the sustainability of current physical activity interventions [24]. Current physical activity interventions are often disrupted due to dependency on professionals, unnatural environments, and high costs [21]. In most cases, access to current physical activity programs is limited to people living in areas where professionals provide services [22]. In fact, in situations such as school closures due to COVID-19 pandemic, children with ASD have completely restricted access to physical activity [55]. Realizing this, researchers have recently begun to show interest in alternative physical activity interventions that can reach more people and that children with ASD can do at home with their families [3]. Various studies have been conducted on sustainable physical activity interventions, especially for children with ASD who cannot access physical activity [21,24,56]. The results of the studies revealed that physical activities provided remotely via WhatsApp and Facebook (Meta) are effective interventions to increase physical activity in children with ASD.

As seen in the literature, alternative physical activity interventions for children with ASD have been focused on social media platforms. Although it is a new topic, no study has been found using ChatGPT to improve the physical activity level of children with ASD. To our knowledge, this is the first study to evaluate the feasibility of ChatGPT-delivered physical activities to increase the physical activity level of children with ASD. In the study, it was taken into consideration that parents have a critical role in the participation of their children with ASD in physical activity [57,58], and parents assumed an important responsibility in the entire process. The parents who participated in the study reported that ChatGPT-delivered physical activities were a feasible and useful intervention to increase the physical activity level of children with ASD. Despite these positive findings, the parents did not understand some of the physical activity content in the WhatsApp group and consulted the researcher about the content. The researcher asked the parents to reprompt ChatGPT regarding the content they did not understand, and the problem was solved in this way. In conclusion, this study demonstrated that ChatGPT-delivered physical activities effectively increased the physical activity level of children with ASD. The findings provided preliminary evidence that ChatGPT-delivered physical activities could be an alternative physical activity that can be easily implemented in the home environment by parents.

### Limitations of the Study

Although the study is a pioneering study examining the effectiveness of ChatGPT-delivered physical activities for children with ASD, it has some limitations. Since the study was conducted as a feasibility study, ChatGPT-delivered physical activities were implemented for only 4 weeks. In addition, a follow-up test was not conducted for the physical activity level of children with ASD. Therefore, it may be difficult to interpret the durability of the effects of ChatGPT-delivered physical activities on the physical activity level of children with ASD. Since the sample consisted of 26 families (parent and child dyads), the generalizability of the survey results regarding the physical activity level of children with ASD may be limited. ChatGPT training was organized to prepare the parents in the intervention group to implement physical activities. However, no interviews were conducted regarding the ChatGPT training to determine the perceived usefulness of this training. We relied on the self-report measures of the parents regarding the diagnoses and physical activity level of the children with ASD who participated in the study, did not make any observations, and excluded the perceptions of the children with ASD about the activity program.

### Implications for Future Research

Future studies may use measurements such as those obtained via observation or pedometers, in addition to the LTEQ, to assess the physical activity levels of children with ASD during ChatGPT-delivered physical activities. This study does not provide any information on comparing ChatGPT-delivered physical activities with other physical activity programs. Future studies may compare the effects of ChatGPT-delivered and face-to-face physical activities. Future studies may include interviews with parents about the benefits of ChatGPT training organized for parents. We used ChatGPT version 4.0 in the study. Since this version only included verbal information, parents did not understand some of the content related to physical activity. Future studies may provide a more understandable process for parents by using different versions of ChatGPT that include image and video support. Furthermore, future studies can use different conversational agents such as BERT, Cipherbot, and DeepSeek to deliver physical activities to parents of children with ASD.

## Abbreviations

ANOVA: analysis of variance
ASD: autism spectrum disorder
LLM: large language model
LTEQ: Leisure Time Exercise Questionnaire
